# Structure of the *Saccharolobus solfataricus* type III-D CRISPR effector

**DOI:** 10.1016/j.crstbi.2023.100098

**Published:** 2023-02-10

**Authors:** Giuseppe Cannone, Dmytro Kompaniiets, Shirley Graham, Malcolm F. White, Laura Spagnolo

**Affiliations:** a MRC Laboratory of Molecular Biology, Cambridge, CB2 0QH, United Kingdom; b School of Molecular Biosciences, College of Medical Veterinary and Life Sciences, University of Glasgow, Glasgow, UK; c School of Biology, University of St Andrews, North Haugh, St Andrews, Fife, KY16 9ST, UK

## Abstract

CRISPR-Cas is a prokaryotic adaptive immune system, classified into six different types, each characterised by a signature protein. Type III systems, classified based on the presence of a Cas10 subunit, are rather diverse multi-subunit assemblies with a range of enzymatic activities and downstream ancillary effectors. The broad array of current biotechnological CRISPR applications is mainly based on proteins classified as Type II, however recent developments established the feasibility and efficacy of multi-protein Type III CRISPR-Cas effector complexes as RNA-targeting tools in eukaryotes. The crenarchaeon *Saccharolobus solfataricus* has two type III system subtypes (III–B and III-D). Here, we report the cryo-EM structure of the Csm Type III-D complex from *S. solfataricus* (SsoCsm), which uses CRISPR RNA to bind target RNA molecules, activating the Cas10 subunit for antiviral defence. The structure reveals the complex organisation, subunit/subunit connectivity and protein/guide RNA interactions of the SsoCsm complex, one of the largest CRISPR effectors known.

## Highlights

• The type III-D CRISPR effector SsoCsm is one of the largest and most complex CRISPR effectors studied to date.
• SsoCsm has a uniquely complex Cas7 backbone structure, which is assembled from seven subunits encoded by five genes.
• The SsoCsm structure reveals conformational flexibility that likely relates to target RNA capture and Cas10 activation.

## Graphical abstract

**Figure:**
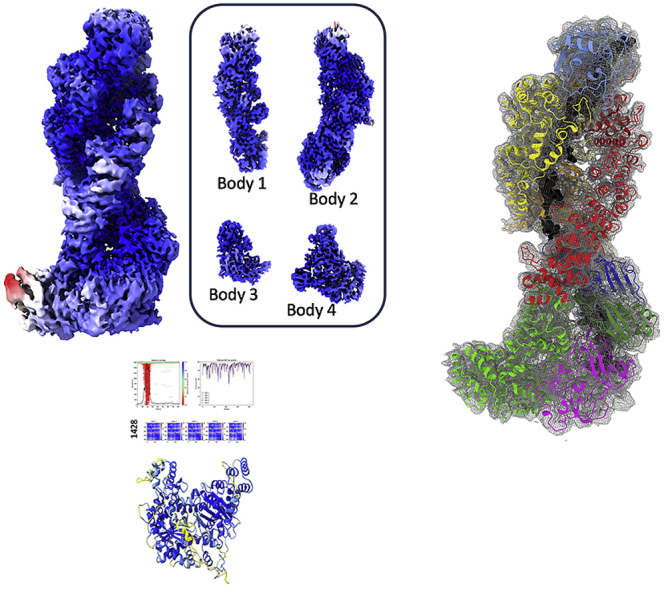

## 1 Introduction

CRISPR effectors are classified in two fundamental classes and six major types: Class 1 (multisubunit, types I, III and IV) and Class 2 (single subunit, types II, V and VI) (Makarova et al., 2015, 2020). Type III CRISPR effectors are evolutionary related to type I systems and both share a CRISPR RNA (crRNA) binding “backbone” made up of Cas7 subunits. They differ in their large subunit: Cas8 for type I and Cas10 for type III (although Type I-D systems are hybrid, with a Cas10 subunit, and type III-E systems lack a Cas10 subunit (Makarova et al., 2020)). Type III CRISPR effectors use their crRNA guide to bind cognate RNA molecules, which typically arise from mobile genetic elements (MGE) such as viruses that have previously been encountered by the CRISPR system. This binding activates the Cas10 subunit, which in different species harbours an HD nuclease domain for ssDNA cleavage, a cyclase domain for cyclic nucleotide synthesis, or a combination of both (reviewed in (Tamulaitis et al., 2017)). The cyclic nucleotides, which are composed of 3–6 AMP subunits linked by 3′, 5’ bonds (henceforth cA_3_, cA_4_ and cA_6_), act as second messengers to activate a wide range of accessory proteins. These include nucleases such as the Csx1/Csm6 family activated by cA_4_ or cA_6_ (Kazlauskiene et al., 2017; Niewoehner et al., 2017), the Can1/Can2/Card1 family activated by cA_4_ (McMahon et al., 2020; Zhu et al., 2021; Rostol et al., 2021) and NucC activated by cA_3_ (Lau et al., 2020; Grüschow et al., 2021). These ancillary nucleases typically have relaxed specificity and target both viral and host nucleic acids to slow down infection or cause abortive infection (Rostol and Marraffini, 2019). The coordination of anti-viral responses at the transcriptional level by cA_4_ are also possible (Charbonneau et al., 2021).

In the past ten years, multiple structural studies have confirmed the overall “boot-shaped” structure of type III effectors, with Cas10 at the toe, Cas5 at the heel and helical constellations of Cas7 and Cas 11 subunits making up the shaft (or backbone) (Rouillon et al., 2013; Spilman et al., 2013; Staals et al., 2013; Benda et al., 2014; Jia et al., 2019; Liu et al., 2019; You et al., 2019; Guo et al., 2019; Smith et al., 2022). The overall organisation resembles that of type I systems, and the two are sometimes known by the collective name “Cascade” (CRISPR-associated complex for antiviral defence) (Brouns et al., 2008). Many groups have contributed to our current understanding of the target RNA recognition and activation of these effectors (reviewed in (Tamulaitis et al., 2017; Athukoralage and White, 2022)), and type III effectors have been repurposed to develop sensitive new diagnostic assays (Grüschow et al., 2021; Steens et al., 2021; Santiago-Frangos et al., 2021). Despite this, the full diversity of type III systems has not been sampled at a structural level, and fundamental questions remain about the mechanism of activation of the Cas10 subunit on target RNA binding (Smith et al., 2022; Wang et al., 2019).

Here, we report the structure of the type III-D (SsoCsm) effector from the thermophilic crenarchaeon *Saccharolobus solfataricus*. SsoCsm was one of the first type III systems studied (Rouillon et al., 2013) and holds the record for the most unique subunits (eight) of any CRISPR effector. The structure of the SsoCsm complex bound to a 48 ​nt crRNA shows its architecture in unprecedented detail. This allows the appreciation of both stoichiometry and connectivity of the complex, which is unique in having a backbone composed of six Cas7-like subunits, encoded by four different genes.

## 2 Materials and methods

The SsoCsm complex was purified from *S. solfataricus* as described previously (Rouillon et al., 2013). Cryo-EM grids were prepared using an FEI Vitrobot Mark IV (Thermo Fisher) at 4 ​°C and 95% humidity. A 4 ​μl volume of SsoCsm complex was applied to holey carbon grids (Quantifoil Cu R1.2/1.3, 300 mesh) covered by a graphene oxide layer (Bokori-Brown et al., 2016), glow-discharged for 45 ​s at a current of 45 ​mA in an EMITECH K100X glow discharger. The grids were then blotted with filter paper once to remove any excess sample, and plunge-frozen in liquid ethane. All cryo-EM data presented here were collected on a ThermoFisher Titan Krios 300 microscope, equipped with a K2 direct detector, located at the eBIC facility. A total of 3907 movies were collected in accurate hole centring mode using EPU. The MotionCorr and GCTF softwares from the Relion 3.1 image processing suite (Zivanov et al., 2018) were used for motion and CTF correction, respectively. Single particle analysis processing was carried out using the Relion 3.1 package, from corrected frames selection, manual particle picking to classification to generate templates for autopicking and subsequent 2D classification and 3D processing. The final reconstruction was obtained from 192,787 particles selected from classes representing both circular and elongated particles at a sampling rate of 1.046 ​Å per pixel and had an overall resolution of 3.52 ​Å, as calculated by Fourier shell correlation at 0.143 cutoff during post-processing. Alphafold2 models (Jumper et al., 2021) built using ColabFold (Mirdita et al., 2022) were generated using the plugin implemented in the ChimeraX package (Pettersen et al., 2021). Individual subunits were fitted in the cryoEM map using the Dock in Map programme within the PHENIX 1.20.1 package (Liebschner et al., 2019). When more than one copy of a given subunit was present, the further subunits were fitted manually to produce a rigid body model of the fully assembled protein component of the complex. After building a model containing all protein subunits, the manual fit was improved with the SegFit routine built in the Chimera package (Pettersen et al., 2021). The RNA molecule was built manually using Coot (Casanal et al., 2020). the coordinates were iteratively refined in real space using the PHENIX Real Space Refinement (Liebschner et al., 2019), Refmac-Servalcat (Yamashita et al., 2021) and Coot (Casanal et al., 2020) packages. Validation was performed using the CryoEM Validation programme in PHENIX 1.20.1 (Liebschner et al., 2019) (Table 1).

**Table 1:** Cryo-EM data collection, refinement and validation statistics.

	SsoCsm, (EMDB-16126), (PDB 8BMW)
**Data collection and processing**	

Voltage (kV)	300
Electron exposure (e−/Å^2^)	32
Defocus range (μm)	0
Pixel size (Å)	1.046
Symmetry imposed	None
Initial particle images (no.)	1,532,976
Final particle images (no.)	192,787
Map resolution (Å)	3.52
FSC threshold	0.143
Map resolution range (Å)	3.411–4.756

Refinement	
Initial model used (PDB code)	Ab initio
**Model**	
Composition (#)	
Chains	15
Atoms	28658 (Hydrogens: 0)
Residues	Protein: 3475 Nucleotide: 48
Water	0
Ligands	0
Bonds (RMSD)	
Length (Å) (# ​> ​4σ)	0.003 (2)
Angles (°) (# ​> ​4σ)	0.537 (13)
MolProbity score	2.01
Clash score	12.06
Ramachandran plot (%)	
Outliers	0.12
Allowed	6.10
Favored	93.78
Rama-Z (Ramachandran plot Z-score, RMSD)	
whole (N ​= ​3455)	0.69 (0.15)
helix (N ​= ​1490)	2.45 (0.14)
sheet (N ​= ​448)	0.20 (0.25)
loop (N ​= ​1517)	−1.57 (0.15)
Rotamer outliers (%)	0.49
Cβ outliers (%)	0.03
Peptide plane (%)	
Cis proline/general	2.7/0.0
Twisted proline/general	0.0/0.0
CaBLAM outliers (%)	4.08
ADP (B-factors)	
Iso/Aniso (#)	
min/max/mean	
Protein	113.15/328.43/164.92
Nucleotide	139.04/201.48/157.56
Ligand	–
Water	–
Occupancy	
Mean	1.00
occ ​= ​1 (%)	100.00
0 < occ <1 (%)	0.00
occ >1 (%)	0.00

Data		
Box		
Lengths (Å)		126.57, 119.24, 242.67
Angles (°)		90.00, 90.00, 90.00
Supplied Resolution (Å)	3.5	
Resolution Estimates (Å)	Masked	Unmasked
d FSC (half maps; 0.143)	–	–
d 99 (full/half1/half2)	4.4/−/−	4.4/−/−
d model	4.0	4.0
d FSC model (0/0.143/0.5)	2.3/3.3/4.0	2.4/3.4/4.0
Map min/max/mean	−0.00/0.07/0.00	

Model vs. Data	
CC (mask)	0.85
CC (box)	0.90
CC (peaks)	0.80
CC (volume)	0.85
Mean CC for ligands	–

## 3 Results and discussion

### 3.1 Cryo-EM structure of SsoCsm

We previously determined the structure of SsoCsm by negative staining TEM (Rouillon et al., 2013). The low resolution of negative staining techniques limited our ability to directly visualise the stoichiometry and connectivity of the subunits, as well as their interaction with the crRNA. We therefore used cryo-EM methods to elucidate the fine detail of this complex, gaining a deeper understanding of its molecular structure. Our previous negative staining and preliminary cryo-EM experiments allowed us to see that the particle distribution was lacking top and bottom views, however this didn't impair the reconstruction of the assembly. On the other hand, cryo grids without a support would lead to preferential side views of the SsoCsm particles. We therefore decided to use graphene oxide (GO) coated grids to emulate the particle distribution typical of the carbon coated grids used in negative staining, at the same time as limiting the background that is typical even of very thin carbon films (Pantelic et al., 2010). Furthermore, in order to improve the particle contrast, which was poor in in-house experiments, we collected data in focus, inserting a Volta phase plate (Danev et al., 2014).

**Fig. 1:**
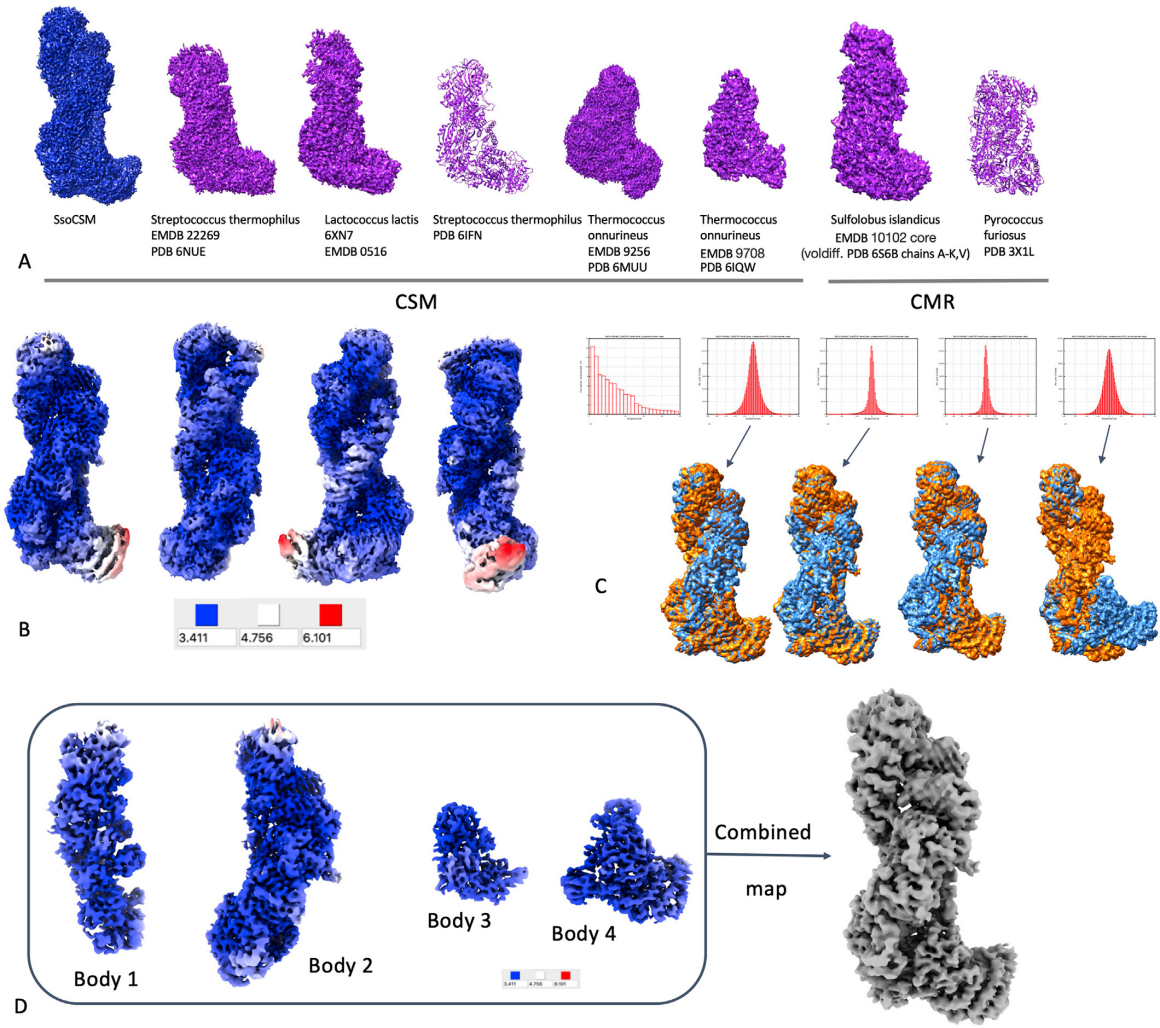
**Structural analysis of SsoCsm.** A. Comparison of the SsoCsm cryo-EM map with other published Csm and Cmr complexes. B. Resolution-filtered SsoCsm map, colour-coded based on local resolution. C. Results of MultiBody analysis, showing histograms for the statistical analysis of eigenvectors 1–4 and volumes for these. The volumes for the two extremes of the analysis are coloured blue and orange, respectively, for eigenvectors 1 to 4. D. 3D reconstructions of bodies 1–4 in the MultiBody analysis, surface coloured based on the local resolution, and full map obtained combining the four bodies in Chimera. (For interpretation of the references to colour in this figure legend, the reader is referred to the Web version of this article.)

We solved the SsoCsm structure at a final overall resolution of 3.52 ​Å (Fig. 1A and supplementary material). The comparison of the SisCmr complex shown in Fig. 1A was edited to exclude the quite unusual Cmr7 subunit (Zhang et al., 2012a), which is present as 13 dimers decorating the crRNP particle (Sofos et al., 2020). In contrast to the SsoCmr complex in negative staining (Zhang et al., 2012b), and other multi-subunit CRISPR systems such as the Type III-A *S. epidermidis* Csm complex (Smith et al., 2022), the SsoCsm complex did not undergo disassembly, showing that it is a rather stable assembly. The analysis of the local resolution of the map (Fig. 1B), showing local resolutions in the range 3.411–6.101 ​Å, suggested that SsoCsm might have some inherent flexibility, particularly with respect to the movement of the Cas10 catalytic subunit, which may be relevant for its activity (Wang et al., 2019). Overall, the structure has common features with other Csm and Cmr complexes, both bacterial and archaeal (Fig. 1A). Poor resolution of the map at the position of the Cas10 subunit, visually shown in the resolution-filtered and colour-coded map shown in Fig. 1B, made it quite difficult to model the entire complex therefore leaving doubts on the conformation of Cas10, in particular its N-terminal half.

A MultiBody refinement experiment allowed the visualization of the main movements within SsoCsm, as shown in Fig. 1C. We decided to group each strand of the double filament in one group (body 1 and body2), while the heel was analysed as body 3 and the tip as body 4. On analysing the output of this four-body refinement, eigenvalues 1 and 2 explain ∼14% and ∼12% of the variance respectively, while eigenvalues 3 and 4 account for around 8% each (Fig. 1C, histograms at the top). The maps at the extremes of the movements are shown in orange and blue in Fig. 1C, lower panel, and movies 1-4 are in the supplementary material. The output from the first eigenvalue analysis highlights an overall swivel movement of body 1 on the long axis of the structure, suggesting an opening to expose the RNA backbone that may be relevant to accommodate target RNA binding. The output from the second analysis highlights an opening of body 2 on the x axis, suggesting an opening towards the crRNA 5′-handle. The third movement is again an opening of body 2, this time on the z axis, again allowing more accessibility towards the 5′ of the RNA molecule bound to the complex. The more complex movement in the fourth analysis is a large rotation ​+ ​translation of the catalytic subunit (body 4), both on the x axis. The latter is likely the component that most affected the anisotropy of the overall reconstruction resolution.

Supplementary video related to this article can be found at https://doi.org/10.1016/j.crstbi.2023.100098

The following are the supplementary data related to this article.

Multimedia component 1 Multimedia component 1

Multimedia component 2 Multimedia component 2

Multimedia component 3 Multimedia component 3

Multimedia component 4 Multimedia component 4

**Fig. 2:**
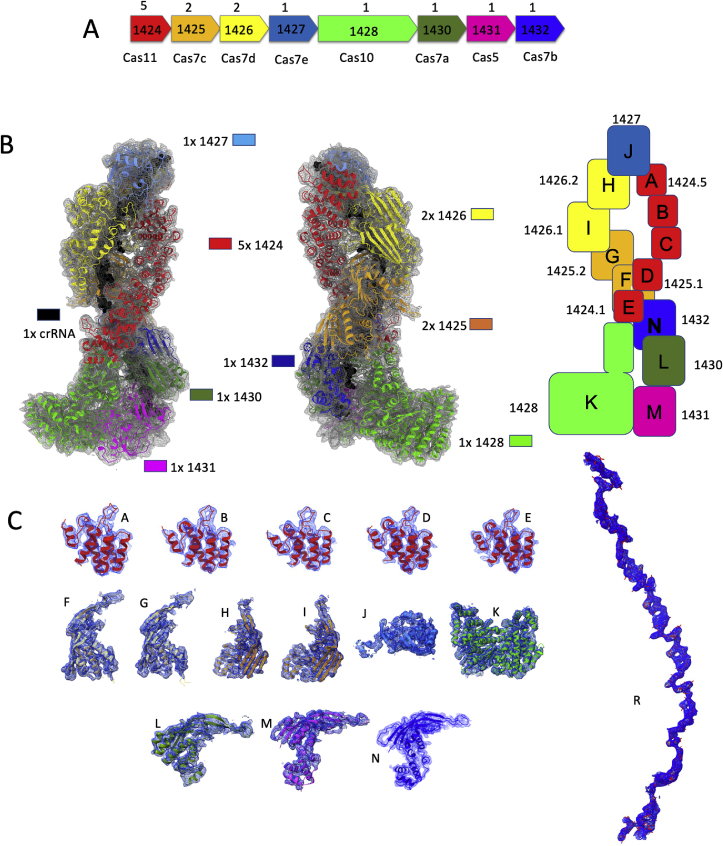
**Interpretation of the SsoCsm cryo-EM structure.** A. SsoCsm operon structure. ORF numbers are shown along their Cas identity and stoichiometry within the complex B. Fitting individual subunit in the cryo-EM map: front and back view are shown, as well as a schematic diagram summarizing position within the complex, stoichiometry and chain name. The RNA molecule (chain R) is shown as black spheres to emphasize its position within the ribonucleoprotein. C. Evaluation of the fit of each chain into the corresponding part of the cryo-EM map.

**Fig. 3:**
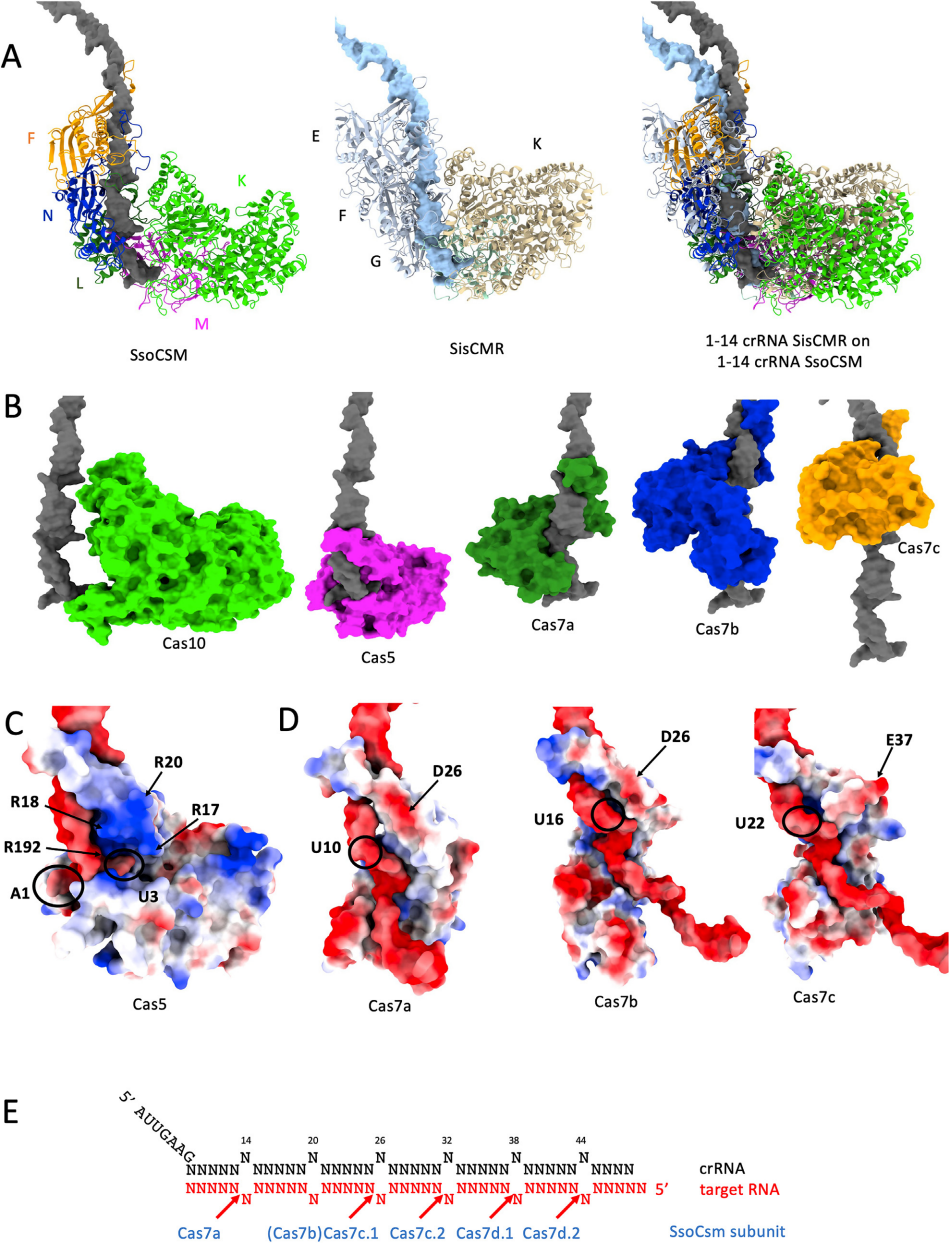
**RNA threading within the SsoCsm cryo-EM structure.** A. Comparison of the 5′ region of crRNA is SsoCsm (this work, pdb 8BMW) and SisCmr (pdb 6S6B). crRNA shown in gray/light blue. B. Position of Cas10, Cas5, Cas7a, Cas7b and Cas7c relative to the crRNA (gray). C. Surface charge distribution and position of catalytically important residues in Cas5, Cas7a, Cas7b and Cas7c relative to the RNA molecule. D. Pattern of crRNA cleavage in SsoCsm. (For interpretation of the references to colour in this figure legend, the reader is referred to the Web version of this article.)

The outputs from the MultiBody experiment in Relion 3.1 (Zivanov et al., 2018) were used to assemble a combined map using the Chimera software (Pettersen et al., 2021). As shown in Fig. 1D, the local resolution of the individual bodies was much more homogeneous, leading to a resolution that would allow confident modelling even for the otherwise blurred catalytic subunit (Fig. 2B and C; Fig. 3B–D). The more detailed combined map assembled in Chimera was used for further fitting experiments, instead of the consensus map.

### 3.2 Fitting ColabFold models, model refinement and analysis

The operon structure of the SsoCsm system, comprising one cas11 gene (1424), one cas5 gene (1431), one cas10 gene (1428), and five cas7-like genes (1425, 1426, 1427, 1430 and 1432), is shown in Fig. 2A. The structures of each SsoCsm subunit were predicted using ColabFold (Mirdita et al., 2022) as built in the ChimeraX software package (Pettersen et al., 2021) (Supplementary Fig. 1). Each model structure had a slightly different coverage and Local Distance Difference Test (lDDT) values (Mirdita et al., 2022; Mariani et al., 2013), with 1432 showing the biggest discrepancies across models.

We fitted one copy of each model in the composite cryoEM map using the *Dock Predicted Model* programme from the PHENIX 1.20.1 package (Liebschner et al., 2019). Subunits present in multiple copies in the complex were assessed by visual inspection of the map and fitted manually. The rigid-body fitted model contained 5 copies of 1424 (chains A-E), 2 copies of 1425 (chain F,G), 2 copies of 1426 (chains H,I), 1 copy of 1427 (chain J), 1 copy of 1428 (chain K), 1 copy of 1430 (chain L), 1 copy of 1431 (chain M) and 1 copy of 1432 (chain N), as well as one 48-ribonucleotide-long RNA chain. These coordinates were refined using the PHENIX Real Space Refinement (Liebschner et al., 2019), Refmac-Servalcat (Yamashita et al., 2021) and Coot (Casanal et al., 2020) packages, to obtain the model shown in Fig. 2B. Fig. 3C shows how each chain within the assembly fits in the corresponding part of the map. Table 1 summarises data collection, processing and refinement data.

This cryo-EM structural analysis yielded a model with an overall stoichiometry of Cas10_1_:Cas5_1_:Cas11_5_:Cas7_7_:crRNA_1_ and a calculated molecular weight of 428 ​kDa, in close agreement with the value of 423 ​kDa estimated by native mass spectrometry (Rouillon et al., 2013). The crRNA is 48 ​nt long, including the 8 ​nt 5′-handle. As the complex was purified from the native host, the bound crRNA is heterogeneous, being sampled from the over 200 spacers present in *S. solfataricus* P1 (Rouillon et al., 2013; Sokolowski et al., 2014), so the 40 ​nt of the spacer have been modelled as uracils. Overall, the structure is reminiscent of that of other Csm and Cmr complexes (some examples are shown in Fig. 1A), formed of two intertwined helical filaments of multiple stacked subunits wrapping around the crRNA, although SsoCsm is the tallest. Reconstitution experiments for SsoCsm (Zhang et al., 2016) showed that each subunit was essential for RNase activity except for the 1427 subunit, which caps the structure (Fig. 2B). Thus, the four different Cas7 subunit types making up the backbone are all essential and cannot substitute for one another.

Comparison with the “core” of SisCmr (pdb 6S6B, chains A-K, V, corresponding to 1xCmr1, 1xCmr2, 1xCmr3, 4xCmr4, 3xCmr5, 1xCmr6) shows that the overall RNA conformation is quite similar, in particular at the 5′-handle (Fig. 3A), and that despite some obvious structural differences in individual subunits the architecture supporting the RNA threading is equivalent in both complexes. The crRNA within SsoCsm has a standard conformation, bound to the intertwined Cas7 filament (Fig. 3). Examination of the foot of the complex shows that there is almost no protein:RNA interaction for the Cas10 subunit, while Cas5 makes tight contacts with the guide RNA molecule (Fig. 3B). These interactions are represented at finer detail in Fig. 3C, which shows that the 5′ handle of the crRNA sits close to a patch of basic residues (R17, 18, 20 and 192) that are clustered together, spatially close to U3. The final nucleotide of the 5′ handle, G8 (sometimes also known as position −1 to discriminate between the handle (−8 to −1) and spacer (1 to X) regions of the crRNA), is flipped, as seen in other type III complexes such as SisCmr (Sofos et al., 2020). Biochemical studies have demonstrated that base pairing with target RNA at this position prevents activation of Cas10 (Kazlauskiene et al., 2016; Johnson et al., 2019; Rouillon et al., 2018).

The remainder of the crRNA adopts a regular pattern with every sixth nucleotide adopting a flipped orientation (Fig. 3). These positions correspond to the sites of cleavage of bound target RNA (Hale et al., 2009), which is catalysed by the Cas7-like backbone subunits (Tamulaitis et al., 2014; Staals et al., 2014; Hale et al., 2014).This activity is important for the dissociation of target RNA and deactivation of the Cas10 subunit (Johnson et al., 2019; Rouillon et al., 2018). Previous studies showed that SsoCsm is unusual in not cleaving at one of the flipped sites, generating a spacing of 12, 6, 6, 6 between sites (Zhang et al., 2016). The missed cleavage corresponds to the Cas7b subunit (1432; chain N in the PDB file, Fig. 3C and D), while subunits competent for RNA cleavage are Cas7a (1430, chain L in the coordinates file), Cas7c (1425, chains F and G) and Cas7d (1426, chains H and I). It is not obvious from this apo structure why Cas7b does not cleave bound target RNA, as it has a similar structure to the other Cas7 subunits and a plausible active site aspartate residue, but this missed cleavage may be due to differences in local target RNA structure.

The diversity of Cas7 subunits in SsoCsm is a unique feature of the complex, as most type III systems make do with just one. It also seems to present some unique challenges for assembly of the complex, which must build in a strict order (from Cas5) with one Cas7a, one Cas7b, two Cas7c and two Cas7d subunits before capping the structure with Cas7e (Fig. 2). The *in vitro* reconstitution experiments reinforce the requirement for each of these subunits in the active complex (Zhang et al., 2016). The two duplicated subunits (7c and 7d) thus make different subunit contacts along the length of the backbone. This might be more easily achieved if these subunits are already dimeric in nature, but this has yet to be confirmed.

### 3.3 Concluding remarks

Here, we have presented the cryo-EM Structure of the type III-D CRISPR effector SsoCsm. With eight different subunits and a molecular weight of 430 ​kDa, this is one of the largest and most complex CRISPR effectors studied to date. A unique feature is the complexity of the Cas7 backbone structure, which is assembled from seven subunits encoded by five genes. Analysis of the structure reveals conformational flexibility that most likely relate to target RNA capture and Cas10 activation. Unfortunately, the inherent diversity of crRNA in this complex precluded analysis of target bound states, but this is a promising area for future study.

## Funding

We acknowledge funding from 10.13039/501100000268BBSRC BB/J005673/1 project grant to LS and MFW and ERC funding to MFW (grant ref 101018608). DK was funded by a 10.13039/501100022719Darwin Trust of Edinburgh grant. We acknowledge Diamond Light Source for access and support of the cryo-EM facilities at the UK's national Electron Bio-imaging Centre (eBIC) under proposal EM16637-14, funded by the 10.13039/100010269Wellcome Trust, 10.13039/501100000265MRC and BBRSC. The Scottish Centre for Macromolecular Imaging (SCMI) is funded by the 10.13039/501100000265MRC (MC_PC_17135) and 10.13039/501100000360SFC (H17007).

## Data accessibility

The SsoCsm composite cryo-EM map was deposited in the EMDB with accession number EMD-16126. Maps for bodies 1, 2, 3 and 4 were deposited under accession numbers EMD-16174, EMD-16175, EMD-16176 and EMD-16177, respectively. The coordinates for the refined model were deposited in the PDB with accession number 8BMW.

## CRediT authorship contribution statement

**Giuseppe Cannone:** Formal analysis. **Dmytro Kompaniiets:** Formal analysis. **Shirley Graham:** Formal analysis. **Malcolm F. White:** Formal analysis, Funding acquisition, Writing – original draft. **Laura Spagnolo:** Formal analysis, Funding acquisition, Writing – original draft.

## Declaration of competing interest

The authors declare that they have no known competing financial interests or personal relationships that could have appeared to influence the work reported in this paper.

## Data Availability

EMDB and PDB entries were deposited and will be made available upon publication. CryoEM data will be deposited on EMPIAR and made available upon publication, too.

## Appendix A Supplementary data

figs1 figs1

figs2 figs2
