# Supplementary figures and images for: Structure of the Saccharolobus solfataricus type III-D CRISPR effector
Source: Curr Res Struct Biol. 2023 Feb 10;5:100098. doi: 10.1016/j.crstbi.2023.100098 (PMC9945777; doi:10.1016/j.crstbi.2023.100098)

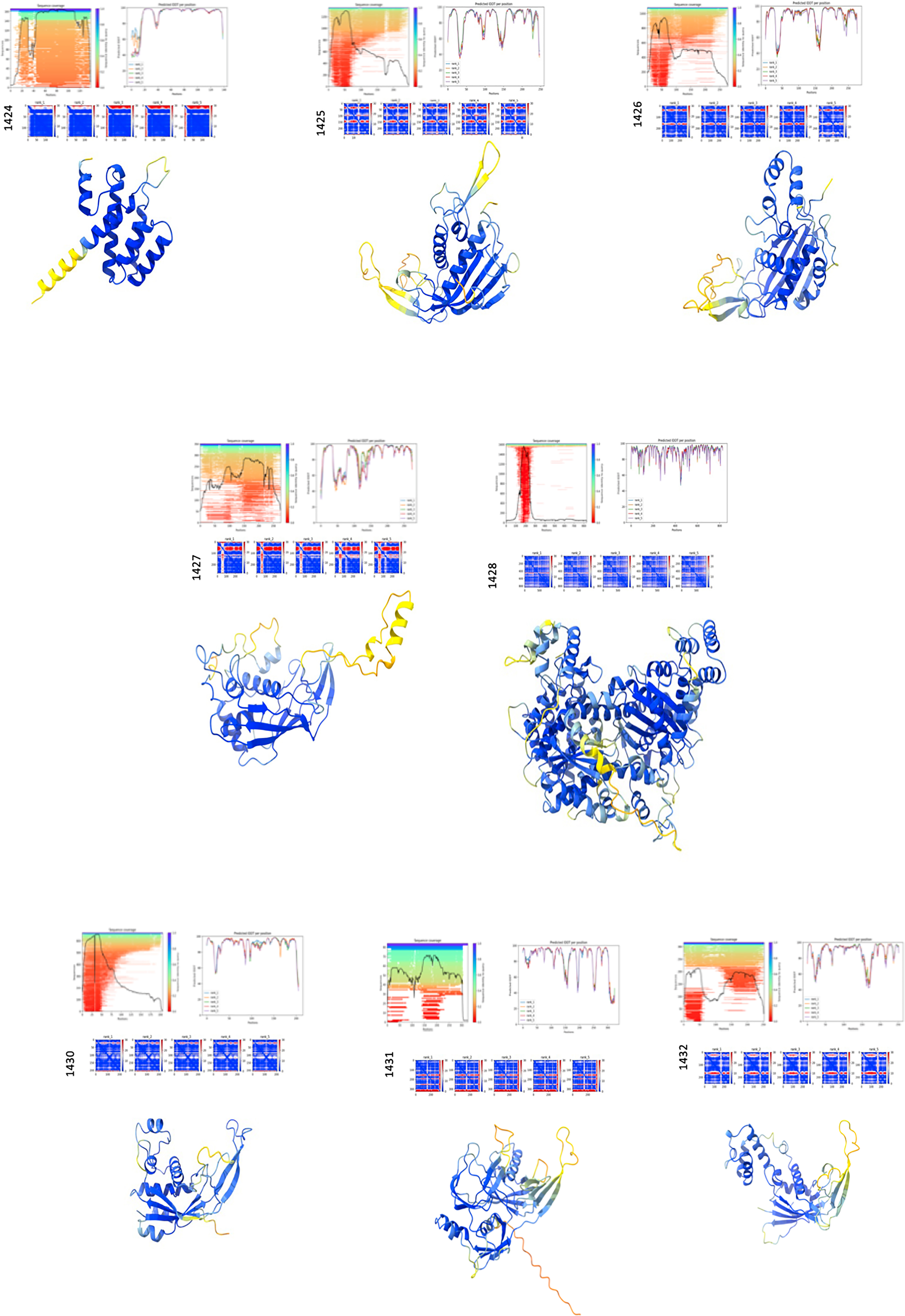

Supplement: figs1 [file figs1.jpg]

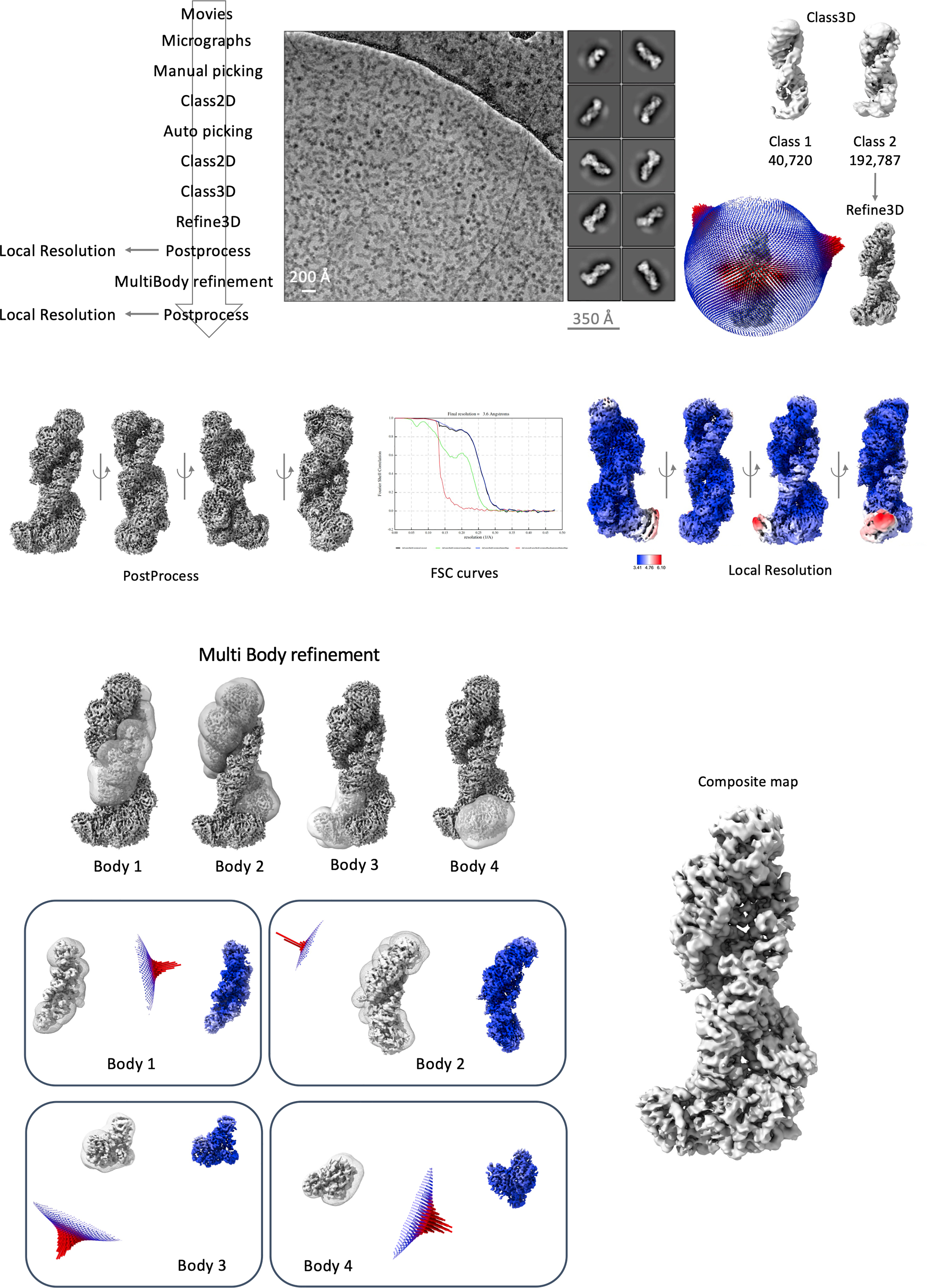

Supplement: figs2 [file figs2.jpg]
